# Two Phenotypes, One Prognosis: Period-Specific Mortality and Prediction of Post-Discharge Events in Acute Heart Failure

**DOI:** 10.3390/jcdd13090423

**Published:** 2026-09-01

**Authors:** Georgios Aletras, Konstantinos Stylianou, Maria Marketou, Maria Stratinaki, Alexandra Papayiannaki, Yannis Pantazis, Michalis Hamilos, Emmanuel Foukarakis

**Affiliations:** 1 Department of Cardiology, Venizelio General Hospital of Heraklion, 71409 Heraklion, Greece; medp2012222@med.uoc.gr (G.A.); maria.stratinaki@gmail.com (M.S.); mfouk@hotmail.com (E.F.); 2 School of Medicine, University of Crete, 70013 Heraklion, Greece; maryemarke@uoc.gr (M.M.); mchamilos@uoc.gr (M.H.); 3 Department of Nephrology, University General Hospital of Heraklion, 71500 Heraklion, Greece; 4 Department of Cardiology, University General Hospital of Heraklion, 71500 Heraklion, Greece; 5 Department of Biochemistry, Venizelio General Hospital of Heraklion, 71409 Heraklion, Greece; alexandrapapayiannaki@gmail.com; 6 Institution of Applied and Computational Mathematics, Foundation for Research and Technology–Hellas (FORTH), 70013 Heraklion, Greece; pantazis@iacm.forth.gr

**Keywords:** acute heart failure, ejection fraction, HFpEF, HFrEF, prognosis, frailty, NT-proBNP, renal replacement therapy

## Abstract

**Background:** The prognostic relevance of left ventricular ejection fraction (LVEF) categories in acute heart failure (AHF) remains debated. We compared the clinical phenotype of patients hospitalized for AHF with preserved (LVEF ≥ 50%) versus reduced (LVEF < 50%) ejection fraction (EF), and compared the prognostic influence of the two phenotypes on in-hospital and post-discharge mortality. **Methods:** We analyzed 530 consecutive patients enrolled in a prospective single-center AHF registry (February 2023–June 2025), followed through June 2026, grouped as preserved (LVEF ≥ 50%, *n* = 264) or reduced (LVEF < 50%, *n* = 266). Baseline characteristics, in-hospital course, and post-discharge events (death, renal replacement therapy [RRT], acute HF rehospitalization, and a triple composite) were compared. Given the distinct prognostic mechanisms operating during hospitalization and after discharge, the two periods were analyzed separately. In-hospital mortality was reported descriptively, whereas predictors of post-discharge mortality were evaluated using multivariable Cox regression with follow-up beginning at discharge. **Results:** Patients with preserved LVEF were older, predominantly women, and more often frail and in atrial fibrillation, whereas LVEF < 50% was associated with an ischemic etiology, right heart failure, and higher values of natriuretic peptides (all *p* < 0.05). In-hospital mortality was higher with LVEF < 50% (7.1% vs. 3.0%, *p* = 0.046) and was almost entirely cardiovascular (6.4% vs. 1.1%, *p* = 0.002). Among the 503 patients discharged alive, post-discharge mortality did not differ between phenotypes (27.7% vs. 22.7%, *p* = 0.23), nor did terminal RRT (2.7% vs. 3.2%) or the triple composite (49.6% vs. 42.1%, *p* = 0.11); emergency-department visits were more frequent with preserved LVEF (69.1% vs. 58.6%, *p* = 0.048). In the multivariable model of post-discharge mortality (*n* = 494, 124 deaths), frailty (HR 1.96, 95% CI 1.09–3.51) and log NT-proBNP (HR 1.86, 95% CI 1.45–2.38) were independent predictors, whereas worsening renal function was not. LVEF < 50% was associated with lower post-discharge mortality in the full model (HR 0.58, 95% CI 0.38–0.89), but this association was not robust across specifications, as follows: it disappeared when NT-proBNP was omitted (HR 0.88, 95% CI 0.60–1.27), and no alternative LVEF cut-point was associated with mortality. **Conclusions:** The two phenotypes are dissimilar in in-hospital mortality and similar in post-discharge mortality, with no consistent independent contribution to prognosis of this specific cut-off of LVEF of 50%.

## 1. Introduction

Heart failure (HF) is traditionally stratified by left ventricular ejection fraction (LVEF) into reduced, mildly reduced, and preserved categories, a framework that guides chronic disease-modifying therapy [1,2]. This framework has since been refined by the Second Universal Definition of Heart Failure, which de-emphasizes rigid ejection-fraction (EF) cut-points in favor of a more integrated, clinically oriented classification and informs the reduced-versus-preserved grouping adopted in the present study [3]. In acute heart failure (AHF), however, the extent to which LVEF category determines prognosis is less certain. Landmark population studies established that patients with preserved EF have a clinical profile distinct from those with reduced EF, yet experience broadly similar mortality [4,5]. Similar crude mortality does not, however, exclude a lower EF attributable risk in the preserved-LVEF population, as follows: after adjustment for age, sex, and clinical risk factors, a survival advantage for preserved EF has been reported [6], so that equal observed mortality may instead reflect a heavier burden of competing determinants. Contemporary registries continue to demonstrate only modest differences in clinical outcomes across the EF spectrum [7,8].

Acute heart failure is increasingly a disease of older, multimorbid, and frail patients, in whom non-cardiac determinants—renal dysfunction, anemia, inflammation, and frailty—may outweigh the influence of EF on outcome [9,10,11,12]. Whether classifying patients with AHF to an LVEF threshold of 50% provides meaningful prognostic discrimination [13], and which variables actually drive risk, has direct implications for how these patients are evaluated and counselled.

In this prospective single-center registry of patients hospitalized with AHF, we sought to (i) characterize and compare patients with preserved (LVEF ≥ 50%) and reduced (LVEF < 50%) EF, (ii) compare in-hospital mortality and clinical outcomes after discharge, and (iii) identify predictors of post-discharge mortality and assess whether the 50% LVEF cut-off contributes prognostic information beyond the accompanying clinical profile.

## 2. Materials and Methods

### 2.1. Study Design and Population

This was a prospective, observational, single-center study conducted at the Department of Cardiology of Venizelio General Hospital, Heraklion, Crete, Greece. Consecutive patients admitted between February 2023 and June 2025 with a primary diagnosis of AHF were enrolled and followed through June 2026. The diagnosis of AHF required the coexistence of clinical signs and symptoms (dyspnea, orthopnea, elevated jugular venous pressure, peripheral oedema, pulmonary crackles) together with objective laboratory and imaging findings (elevated natriuretic peptides, evidence of structural cardiac disease, dilated inferior vena cava, B-lines on lung ultrasound), in accordance with the 2021 European Society of Cardiology (ESC) Heart Failure Guidelines. Elevated natriuretic peptides were defined as NT-proBNP ≥ 300 pg/mL, consistent with the acute-setting threshold of the 2021 ESC Guidelines [1]; because natriuretic-peptide concentrations are lower in patients with obesity and may be higher in older patients and in those with atrial fibrillation or chronic kidney disease, values were interpreted in the context of body habitus, age, and these comorbidities. Patients younger than 18 years, those already receiving renal replacement therapy (RRT), and those admitted primarily for ST-elevation myocardial infarction or an acute coronary syndrome without a dominant AHF presentation were excluded. The final analysis cohort comprised 530 patients with complete one-year follow-up. All participants provided written informed consent; the study was approved by the Scientific Council of Venizelio General Hospital of Heraklion (5 August 2022; protocol 15/05-08-2022) and conducted in accordance with the Declaration of Helsinki.

### 2.2. Grouping

Patients were divided by transthoracic-echocardiographic LVEF into a preserved group (LVEF ≥ 50%; *n* = 264) and a reduced group (LVEF < 50%; *n* = 266). Consistent with the Second Universal Definition of Heart Failure, EF below 50% was analyzed as a single reduced-LVEF category [3]; for brevity this group is hereafter designated the ‘LVEF < 50%’ group. A three-level categorization (preserved ≥50%, 41–49%, ≤40%) was retained for sensitivity analyses (Supplementary Table S1).

### 2.3. Data Collection

At admission, demographic characteristics (age, sex), primary HF etiology (ischemic, valvular, hypertensive, or other), de novo versus acute-on-chronic status, New York Heart Association (NYHA) functional class, and a comprehensive comorbidity profile were recorded. Comorbidities included coronary artery disease, arterial hypertension, atrial fibrillation (AF), chronic kidney disease (CKD), diabetes mellitus, active or prior malignancy, airway disease, stroke/transient ischemic attack, active smoking, and prior device therapy or revascularization. Admission laboratory values included creatinine, estimated glomerular filtration rate (eGFR), albumin, sodium, potassium, urea, C-reactive protein, N-terminal pro–B-type natriuretic peptide (NT-proBNP), high-sensitivity troponin I, transaminases, lipid profile, red-cell distribution width, hemoglobin, and white-cell count; peak in-hospital creatinine and nadir eGFR were also recorded. Chronic and discharge HF pharmacotherapy (renin–angiotensin inhibitors/angiotensin receptor–neprilysin inhibitors [ARNI], beta-blockers, mineralocorticoid-receptor antagonists [MRA], sodium–glucose cotransporter-2 inhibitors [SGLT2i] and diuretics) was documented.

### 2.4. Definitions

Right heart failure was defined by the coexistence of at least two of the following: (i) clinical systemic venous congestion (elevated jugular venous pressure, peripheral oedema, hepatomegaly, or ascites) without a predominant left-sided cause; (ii) echocardiographic right ventricular (RV) dilation and/or systolic dysfunction (tricuspid annular plane systolic excursion [TAPSE] < 17 mm and/or tricuspid annular S′ < 9.5 cm/s); and (iii) elevated right-sided pressures (estimated pulmonary artery systolic pressure [PASP] >35 mmHg or moderate-to-high echocardiographic probability of pulmonary hypertension); isolated right HF denoted right HF without significant left-sided dysfunction.

Coronary artery disease was defined as ≥50% stenosis in at least one epicardial vessel or equivalent evidence of ischemia on non-invasive testing. CKD was defined as eGFR < 60 mL/min/1.73 m^2^ persisting for ≥3 months, staged by the Kidney Disease: Improving Global Outcomes (KDIGO) 2012 classification [14] (Supplementary Table S2). Frailty was assessed with the Clinical Frailty Scale (CFS) [15] according to the patient’s baseline pre-decompensation status (non-frail 1–3, vulnerable/mildly frail 4–5, moderately-to-severely frail 6–9), with CFS ≥ 5 defining frailty (Supplementary Table S3).

Acute kidney injury (AKI) was defined as in-hospital increase in serum creatinine of ≥0.3 mg/dL within 48 h or to ≥1.5-fold from baseline, in accordance with KDIGO criteria [16]. Worsening renal function (WRF) was graded as an ordinal class from the ratio of peak to baseline serum creatinine (class 0, <1.25; 1, 1.25–1.50; 2, 1.51–2.00; 3, 2.01–3.00; 4, >3.00), with in-hospital renal replacement therapy constituting the most severe grade (class 5) (Supplementary Table S4).

Clinical phenotypes at presentation (acute decompensated HF, acute pulmonary oedema, isolated right HF, cardiogenic shock) (Supplementary Table S5) and significant (moderate or severe) valvular disease were classified according to the 2021 ESC Guidelines and ESC/EACVI recommendations [1,17]; multivalvular disease denoted at least moderate disease in more than one valve.

### 2.5. Echocardiographic Assessment

Transthoracic echocardiography was performed during the index hospitalization, after initial clinical stabilization (at a mean of 4 days after admission), and interpreted by two separate experienced cardiologists. LVEF was measured by Simpson’s biplane method [18]. Right ventricular systolic function was assessed by TAPSE and tricuspid annular S′ velocity, left ventricular diastolic function by the E/e′ ratio, and left atrial volume index was measured and indexed to body surface area. Estimated PASP was derived from the peak tricuspid regurgitation velocity and estimated right atrial pressure. The echocardiographic probability of pulmonary hypertension was graded according to the 2022 ESC/ERS criteria [19] (Supplementary Table S6).

### 2.6. Follow-Up and Endpoints

Patients were followed for 12 months through outpatient clinic visits or structured telephone contact. The primary endpoint was all-cause death, analyzed separately for the in-hospital period and for the period beginning at discharge; the secondary endpoint was need for terminal RRT. Emergency-department (ED) visits, all-cause and acute-HF-specific rehospitalizations, and a composite endpoint of death, acute HF rehospitalization, or RRT were also recorded. Death and terminal RRT were treated as cumulative events ascertained in all patients discharged alive; a patient reaching RRT was counted as an event irrespective of subsequent death, and for analyses of RRT, death without prior RRT was not considered an RRT event. The RRT endpoint denotes terminal renal replacement therapy, that is, dialysis initiated and maintained as a permanent modality; a transient in-hospital dialysis episode followed by recovery of renal function was recorded as in-hospital RRT but was not counted as an RRT endpoint or as a component of the composite. One patient met this description. Non-fatal recurrent events (rehospitalization, ED visits) were assessed among patients alive and free of RRT during the relevant interval, as those reaching the primary or secondary endpoint were no longer at risk. The binary follow-up field for these endpoints was completed only for patients who remained alive and free of terminal RRT, so events occurring in patients who subsequently died are absent from that field; the proportions reported for individual non-fatal endpoints therefore refer to that denominator and are not first-event rates. For acute HF rehospitalization, the timing of the event was nevertheless retained in the composite event-time variables, which allowed the order of events to be reconstructed. The triple composite, by contrast, was defined in the whole cohort as the first occurrence of death, acute HF rehospitalization, or RRT. To address the resulting bias, the cumulative incidence of first acute HF rehospitalization was additionally estimated with death and terminal RRT as competing events using the Aalen–Johansen estimator, the order of events being determined by comparing the time to the first composite event with the time to death or terminal RRT, both recorded in weeks, so that a patient whose first composite event preceded death or RRT was classified as having been rehospitalized first. This recovered 57 first rehospitalizations occurring in patients who subsequently died; groups were compared by Gray’s test and by a Fine–Gray subdistribution model. Mortality was ascertained for the entire cohort. In-hospital deaths were additionally adjudicated as cardiovascular or non-cardiovascular by two cardiologists from the complete clinical record; cause of death could not be reliably adjudicated for deaths occurring after discharge, and post-discharge mortality is therefore reported as all-cause only.

### 2.7. Statistical Analysis

Continuous variables were assessed for normality (Shapiro–Wilk) and presented as median (interquartile range [IQR]) or mean ± standard deviation (SD), compared with the Mann–Whitney U or *t*-test accordingly; categorical variables were presented as *n* (%) and compared with the χ^2^ or Fisher’s exact test. Time-to-event analyses used Kaplan–Meier estimation with the log-rank test. Ιn-hospital and post-discharge mortality reflect distinct prognostic processes and should not be assumed to share a common hazard structure. Moreover, WRF and cardiogenic shock are identified during the index hospitalization and therefore cannot be treated as baseline covariates in a model beginning at admission. Mortality was consequently analyzed separately for the in-hospital and post-discharge periods. In-hospital mortality was described but not modeled, given only 27 events. Post-discharge mortality, with time zero at discharge, was the sole endpoint for multivariable modeling, and was analyzed by Cox proportional-hazards regression in the 503 patients discharged alive. Because follow-up ended 12 months after admission, the post-discharge observation window varied with length of stay (median 51.0, range 44.7–51.7 weeks); survivors were censored at their individual end of follow-up rather than at a common 12-month point. Covariates were selected a priori on the basis of established prognostic relevance in AHF and prior literature, constrained by the available number of events (approximately 10 outcome events per candidate predictor); no automated or stepwise variable selection procedures were used. The covariates were LVEF category, age, sex, frailty, log NT-proBNP, baseline eGFR, hemoglobin, sodium, and WRF class. Worsening renal function was modeled as the ordinal WRF class, whose most severe grade incorporates in-hospital RRT. Cardiogenic shock could not be retained, since only one of the six patients presenting in shock survived to discharge. The model was fitted using complete case analysis (*n* = 494), because of missing data in a single predictor (NT-proBNP); as a sensitivity analysis, missing values were handled by multiple imputation by chained equations (m = 10 imputations) with pooling by Rubin’s rules. Model discrimination was summarized by Harrell’s C-index.

To examine whether the prognostic structure differed by LVEF category, the model was fitted separately within each LVEF group among hospital survivors and corresponding coefficients were compared by a Wald test of their difference, discrimination being compared between strata; this stratified approach was preferred to fitting multiplicative interaction terms in the full model, which proved unstable given the number of events. Because the dichotomy at 50% is conventional, alternative LVEF cut-points (40%, 45%, and 55%) were examined in the same post-discharge model as a sensitivity analysis. Within the preserved-LVEF group, the prognostic contribution of individual clinical and echocardiographic substrates was additionally examined in exploratory Cox models adjusted for age, frailty, and log NT-proBNP. Subgroup and sensitivity analyses were exploratory and were not adjusted for multiple comparisons. Competing-risks analysis of first acute HF rehospitalization, with death and terminal RRT as competing events, was performed in R 4.6.0 (R Foundation for Statistical Computing, Vienna, Austria) using the cmprsk package: cumulative incidence functions and Gray’s test with the cuminc function, and the Fine–Gray subdistribution hazard model with the crr function. All other analyses were performed in Python 3.12 (Python Software Foundation, Wilmington, DE, USA) using lifelines 0.27.8, pandas 2.2.1, NumPy 1.26.4, SciPy 1.12.0, scikit-learn 1.4.0, and Matplotlib 3.10.8. A two-sided *p* < 0.05 was considered significant.

## 3. Results

### 3.1. Baseline Characteristics

Of the 530 patients, 264 (49.8%) had LVEF ≥ 50% and 266 (50.2%) LVEF < 50%. The two groups differed substantially in their baseline demographic and clinical characteristics (Table 1). Patients with preserved LVEF were older (median 82 vs. 79 years, *p* < 0.001) and predominantly women (61.4% vs. 31.6%, *p* < 0.001), with a higher prevalence of AF (67.8% vs. 50.4%, *p* < 0.001) and frailty (CFS ≥ 5: 73.5% vs. 59.4%, *p* < 0.001), whereas patients with LVEF < 50% more often had an ischemic etiology (55.3% vs. 7.6%, *p* < 0.001) and right HF (47.4% vs. 37.9%, *p* = 0.034). Hypertension was numerically more common in the preserved group (89.8% vs. 84.6%, *p* = 0.098). Heart-failure pharmacotherapy is summarized in Table 2 and, as expected, discharge prescription of guideline-directed disease-modifying therapy was more frequent in the LVEF < 50% group. Admission laboratory and echocardiographic findings are summarized in Table 3. Patients with LVEF < 50% had higher NT-proBNP and high-sensitivity troponin I concentrations, as well as higher hemoglobin levels (12.1 vs. 11.4 g/dL, *p* = 0.002). In-hospital course and outcomes are summarized in Table 4; the distribution of WRF classes during hospitalization was similar between groups (*p* = 0.79).

### 3.2. In-Hospital Course and Post-Discharge Outcomes

**Figure 1:**
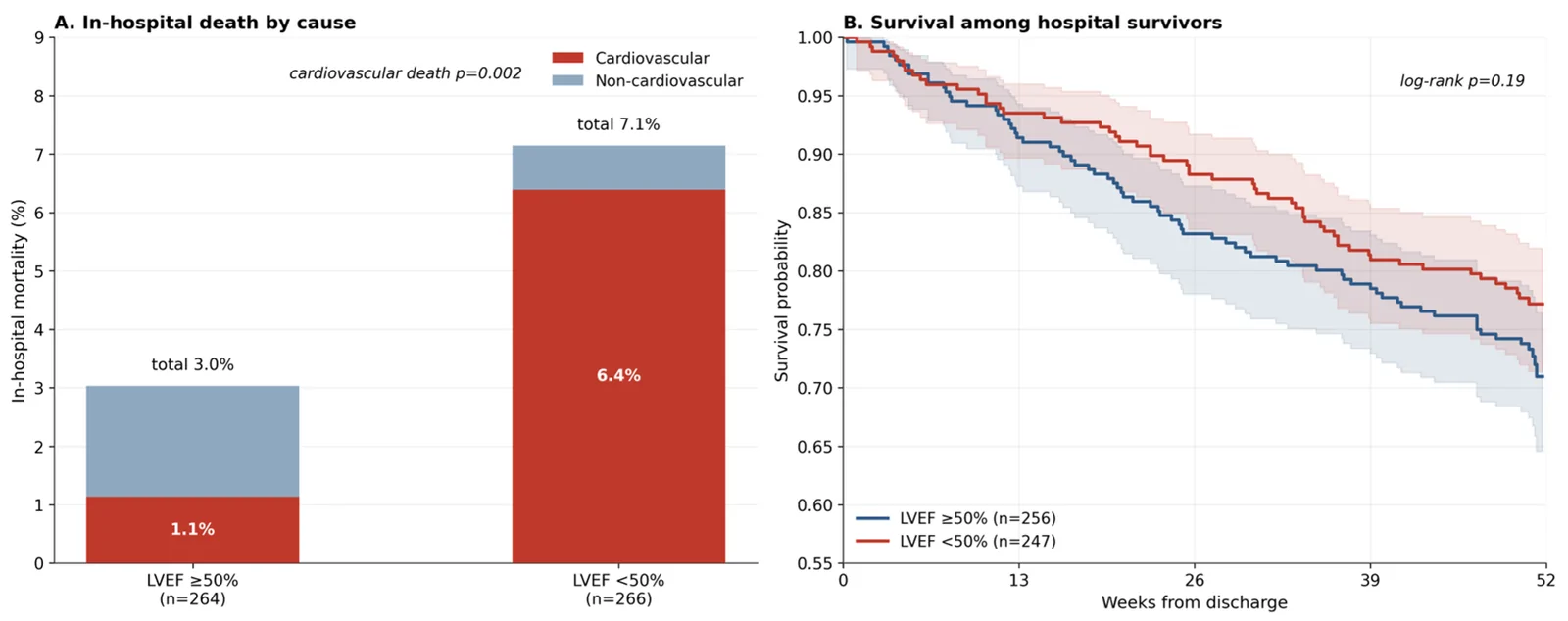
Temporal and cause-specific separation of mortality by EF. (**A**) In-hospital mortality partitioned into cardiovascular and non-cardiovascular death; the excess in the LVEF < 50% group was almost entirely cardiovascular (6.4% vs. 1.1%, *p* = 0.002), whereas non-cardiovascular in-hospital death did not differ. (**B**) Kaplan–Meier survival among the 503 patients discharged alive, with time zero at discharge; the survival curves did not differ significantly (log-rank *p* = 0.19). Shaded areas are 95% confidence intervals. Abbreviations: LVEF, left ventricular ejection fraction.

**Figure 2:**
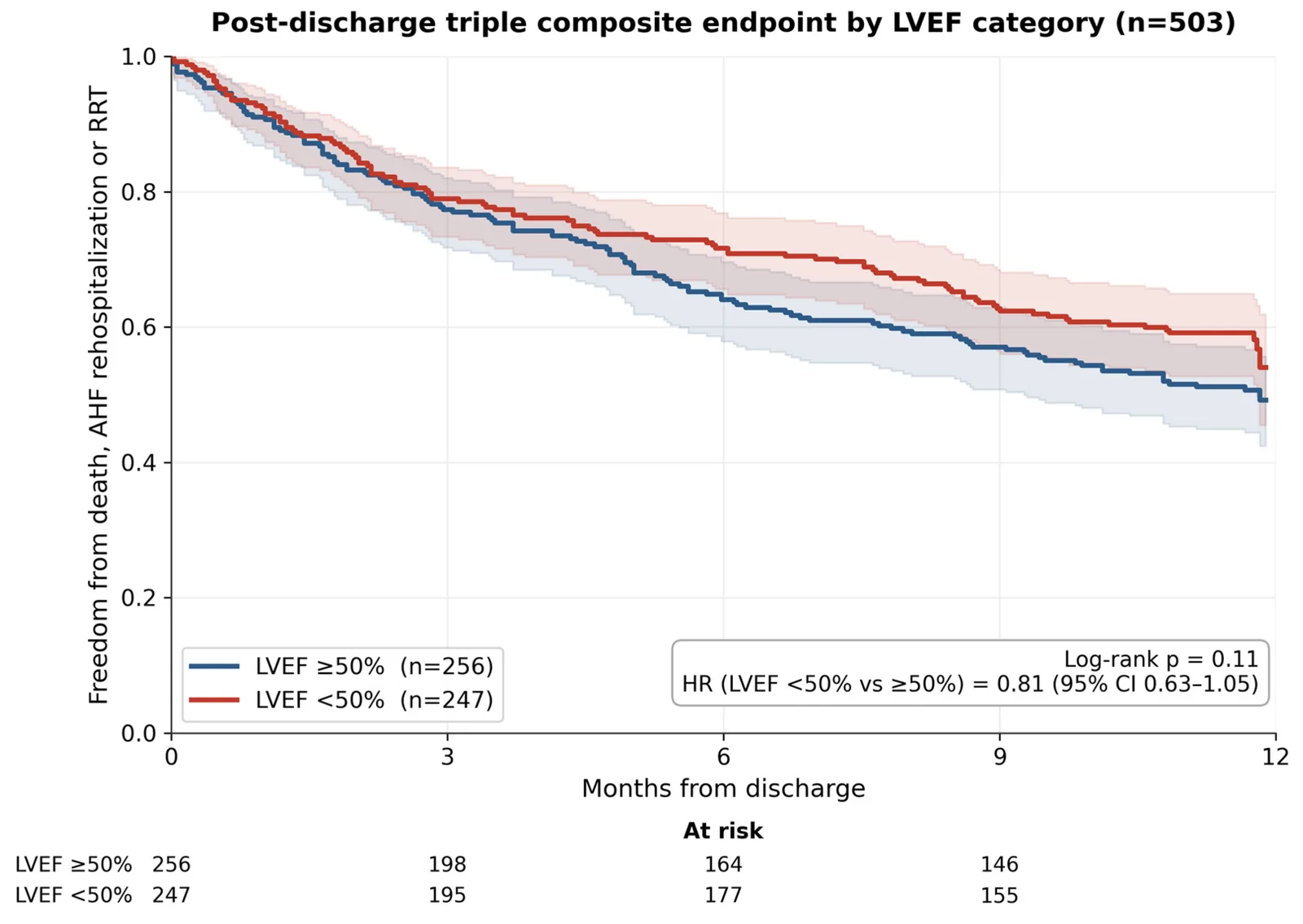
Kaplan–Meier freedom from the post-discharge triple composite endpoint (death, acute HF rehospitalization, or renal replacement therapy) by LVEF category, in the 503 patients discharged alive. Time zero is discharge; follow-up ended 12 months after admission, so the observation window varied with length of stay. Shaded areas are 95% confidence intervals. Abbreviations: AHF, acute heart failure; HR, hazard ratio; LVEF, left ventricular ejection fraction; RRT, renal replacement therapy.

In-hospital mortality was lower in the preserved group (3.0% vs 7.1%, *p* = 0.046) (Table 4). This difference was almost entirely attributable to cardiovascular death, as follows: 17 of the 19 in-hospital deaths in the LVEF < 50% group were cardiovascular (6.4% of the group) compared with 3 of 8 in the preserved group (1.1%; *p* = 0.002), whereas non-cardiovascular in-hospital death did not differ (2 [0.8%] vs. 5 [1.9%], *p* = 0.28) (Figure 1A). Overall one-year mortality was 29.1% and did not differ significantly between phenotypes (29.9% vs. 28.2%, *p* = 0.70); this similar overall figure, however, combined a significant difference during the index hospitalization with no significant difference thereafter (Table 4). Among the 503 patients discharged alive, the two phenotypes no longer differed. Post-discharge mortality was 27.7% with preserved and 22.7% with reduced LVEF (*p* = 0.23; Figure 1B), terminal RRT occurred in 2.7% and 3.2% (*p* = 0.94) (Table 5), and the triple composite of death, acute HF rehospitalization, or RRT affected 49.6% and 42.1% (*p* = 0.11) (Table 6). In survivor-only descriptive analyses, acute HF rehospitalization did not differ (28.3% vs. 23.1%, *p* = 0.28), whereas emergency-department visits were more frequent in the preserved group (69.1% vs. 58.6%, *p* = 0.048). Kaplan–Meier analysis of the post-discharge composite endpoint showed no significant difference between groups (log-rank *p* = 0.11; hazard ratio (HR) for LVEF < 50% vs. ≥ 50% 0.81, 95% confidence interval (CI) 0.63–1.05) (Figure 2). When first acute HF rehospitalization was analyzed with death and terminal RRT as competing events, and the order of events was recovered from the composite event times, 151 patients experienced a first rehospitalization; the 12-month cumulative incidence was 31.4% with preserved and 25.6% with reduced LVEF (Gray’s test *p* = 0.13; subdistribution hazard ratio 0.78, 95% CI 0.57–1.08) (Table 6).

### 3.3. Predictors of Post-Discharge Mortality

**Figure 3:**
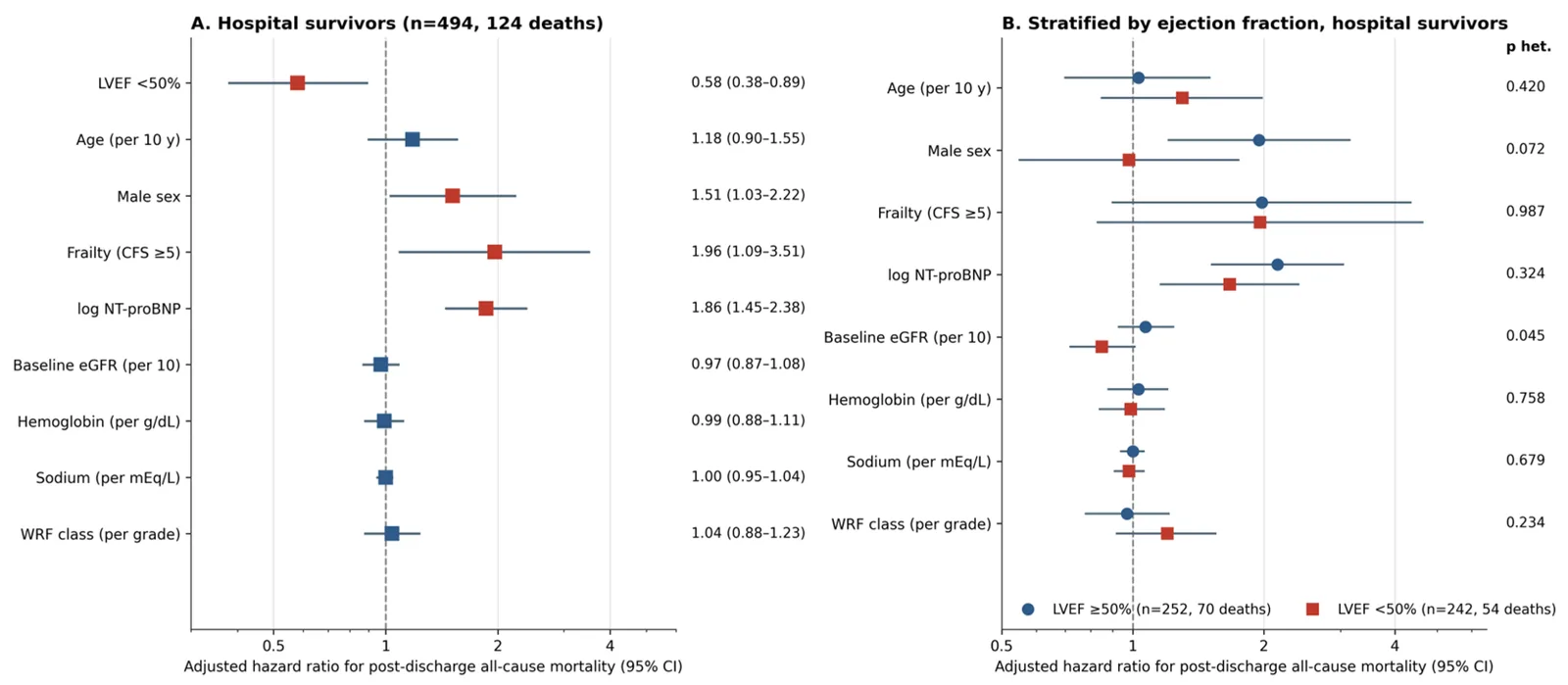
Adjusted hazard ratios for post-discharge all-cause mortality. (**A**) Multivariable model with time zero at discharge, fitted in the 494 of 503 patients discharged alive who had complete data for all covariates (124 deaths). Cardiogenic shock was not estimable in this population and is omitted. Red markers denote associations significant at *p* < 0.05. (**B**) The same model fitted separately within each LVEF stratum (252 and 242 patients with complete data, 70 and 54 deaths), with the Wald *p* value for heterogeneity of each coefficient between strata shown on the right. No covariate showed heterogeneity surviving correction for multiple testing. Abbreviations: CFS, Clinical Frailty Scale; CI, confidence interval; eGFR, estimated glomerular filtration rate; LVEF, left ventricular ejection fraction; NT-proBNP, N-terminal pro–B-type natriuretic peptide; WRF, worsening renal function.

The multivariable model was fitted in the 503 patients discharged alive, with time zero at discharge (*n* = 494 with complete data, 124 deaths; C-index 0.710). Cardiogenic shock could not be evaluated, because only one of the six patients presenting in shock survived to discharge. Frailty (HR 1.96, 95% CI 1.09–3.51, *p* = 0.025) and log NT-proBNP (HR 1.86, 95% CI 1.45–2.38, *p* < 0.001) were independent predictors of post-discharge mortality, whereas worsening renal function showed no independent association (HR 1.04 per grade, 95% CI 0.88–1.23, *p* = 0.62) (Table 7, Figure 3A). LVEF < 50% was associated with lower post-discharge mortality in this model (HR 0.58, 95% CI 0.38–0.89, *p* = 0.012), but the association was not robust across model specifications, as follows: it disappeared when NT-proBNP was omitted (HR 0.88, 95% CI 0.60–1.27, *p* = 0.49) and in a model adjusted for age and sex alone (HR 0.84, 95% CI 0.58–1.22, *p* = 0.37), and no alternative cut-point was associated with mortality (<40%: HR 0.79, *p* = 0.30; <45%: HR 0.73, *p* = 0.16; <55%: HR 0.84, *p* = 0.41) (Supplementary Table S7). Because NT-proBNP is closely related both to EF and to clinical severity, the adjusted LVEF coefficient was sensitive to model specification and should not be interpreted as a robust independent prognostic effect.

Missing data were confined to a single variable. Among the 503 patients discharged alive, NT-proBNP was unavailable in 9 (1.8%), of whom 3 died during follow-up; all remaining covariates were complete, so 494 patients (124 deaths) contributed to the model. Multiple imputation gave results essentially identical to the complete-case analysis (LVEF < 50%: HR 0.78, 95% CI 0.53–1.13).

### 3.4. Subanalysis of Post-Discharge Mortality in the Two Phenotypes

Because interaction models including a full set of multiplicative terms were unstable given the number of events, homogeneity of effect was assessed by fitting the model separately within each LVEF stratum among hospital survivors (252 and 242 patients, 70 and 54 deaths) and comparing corresponding coefficients (Table 7, Figure 3B). Seven of the eight covariates showed no heterogeneity between phenotypes (*p* for heterogeneity 0.072 to 0.987). The only comparison reaching nominal significance was baseline eGFR (1.07 per 10 mL/min/1.73 m^2^ in preserved versus 0.85 in reduced EF, *p* = 0.045); as one of eight uncorrected comparisons, it does not survive correction for multiple testing (Bonferroni-corrected *p* = 0.36) and is not interpreted further. Log NT-proBNP predicted mortality in both strata (HR 2.15 and 1.67), and frailty was almost identical (1.98 and 1.96). Discrimination was lower in the preserved-LVEF model (C-index 0.693) than in the LVEF < 50% model (0.751). With 70 and 54 deaths, respectively, these analyses are exploratory, and the prognostic architecture appears essentially shared between the two phenotypes.

## 4. Discussion

### 4.1. Principal Findings

In this prospective registry of 530 consecutive patients hospitalized for AHF, preserved and reduced (LVEF < 50%) EF identified two clinically distinct populations whose prognosis differed during the index hospitalization but not thereafter. In-hospital mortality was more than twice as high with reduced EF and was almost exclusively cardiovascular. Among the 503 patients discharged alive, neither all-cause death, terminal RRT, nor the triple composite differed between phenotypes; the cumulative incidence of first acute HF rehospitalization was numerically higher with preserved EF, in the same direction as the excess of emergency-department visits, but did not reach statistical significance. On multivariable analysis, post-discharge mortality was driven by natriuretic-peptide levels and frailty rather than by EF. The prognostic association of the 50% LVEF cut-off was not robust across model specifications, and no alternative cut-off was independently associated with outcome. Worsening renal function was not independently associated with post-discharge mortality, whereas cardiogenic shock could not be evaluated, because only one of the six affected patients survived to discharge; the prognostic architecture was otherwise shared between the two phenotypes.

### 4.2. Distinct Phenotypes Consistent with Contemporary Registries

The phenotypic contrast we observed is consistent with the well-established dichotomy between heart failure with preserved (HFpEF) and reduced (HFrEF) EF. Because the two groups were defined a priori by EF, these comparisons characterize the phenotypes rather than test whether they differ. Patients with preserved LVEF were older, predominantly women, and more frequently had AF, whereas those with LVEF < 50% were more often men with an ischemic etiology, higher natriuretic peptides and troponin, and more right heart involvement. These distinctions mirror the large ESC-HFA EORP Heart Failure Long-Term Registry, in which acute HF with preserved, mildly reduced, and reduced EF differed analogously in sex, etiology, and comorbidity distribution [7], and are consistent with foundational population studies [4,5] and the chronic ESC-HF Long-Term Registry [8]. The high burden of frailty among our preserved-LVEF patients aligns with the increasingly recognized overlap between HFpEF and an older, multimorbid, and frail geriatric phenotype [7,9].

Beyond these classical descriptors, the preserved-LVEF group was distinguished by its valvular and pulmonary vascular substrate, as follows: at least moderate tricuspid regurgitation (49.6% vs. 38.0%, *p* = 0.009) and mitral stenosis (6.1% vs. 2.3%, *p* = 0.048) were more frequent, as was a high echocardiographic probability of pulmonary hypertension (59.1% vs. 47.0%, *p* = 0.022), whereas at least moderate mitral regurgitation was more frequent with LVEF < 50% (50.0% vs. 35.6%, *p* = 0.001) and multivalvular disease was equally distributed (34.1% vs. 35.7%). These findings characterize a preserved-LVEF phenotype marked by right-sided and valvular congestion alongside substantial renal and vascular comorbidity, rather than by left ventricular systolic dysfunction. After adjustment for age, frailty, and NT-proBNP, none of these features was independently associated with prognosis, except for an inverse association with a history of hypertension that likely reflects residual confounding (Supplementary Table S8).

A formal phenotypic characterization of preserved-ejection-fraction heart failure, including its obesity-related, valvular, and pulmonary vascular subtypes, requires anthropometric and invasive hemodynamic data that were not collected in this registry.

### 4.3. Comparable Outcomes Across the EF Spectrum

Despite these phenotypic differences, post-discharge all-cause mortality, terminal RRT, and the triple composite did not differ across LVEF categories. This is concordant with prior evidence, as follows: the MAGGIC meta-analysis demonstrated only a modest survival advantage for preserved EF in chronic HF [13], and contemporary acute-HF and Asian registries have reported broadly similar mortality across the EF spectrum, with mildly reduced EF often occupying an intermediate or even favorable position [7,20,21,22]. Our finding that one-year mortality was essentially identical (28–30%) and that the triple composite affected nearly half of both groups extends this observation to an older, real-world Mediterranean acute-HF cohort. Similar observed mortality at the population level should not, however, be read as therapeutic equivalence. SGLT2i reduce mortality in reduced EF but act predominantly on hospitalization in preserved EF [23]; the contribution of pharmacotherapy to overall prognosis in an elderly multimorbid population is modest, and treatment effects that apply to only one phenotype cannot be inferred from the absence of an ejection-fraction effect on outcome. Non-fatal events showed a similar, albeit non-significant, pattern, but the cohort was too small to determine whether fatal and non-fatal outcomes genuinely diverged.

Background pharmacotherapy in this cohort reflects a period of transition in guideline implementation. Before admission, only 20.4% of patients were receiving an SGLT2i (14.8% with preserved and 25.9% with reduced EF) and 3.8% an ARNI; by discharge SGLT2 inhibitor use had risen to 64.2% overall, though it remained lower in preserved EF (53.5% vs. 75.3%). Adoption was influenced by the evolving guideline landscape and limited by advanced age, frailty, chronic kidney disease, and the lack of an established indication for ARNI in patients with preserved EF. These rates nonetheless compare favorably with contemporary practice elsewhere, as follows: among 158,849 patients hospitalized in the United States with an EF above 40% between 2021 and 2023, only 13.9% were discharged on an SGLT2i, with the same preserved-versus-mildly-reduced gradient we observed (13.0% vs. 18.5%) [24]; nationwide data confirm persistent delayed initiation and under-titration after an AHF admission [25]. The preserved-LVEF treatment gap appears to be a general rather than a purely local phenomenon, and the substantial in-hospital uptake indicates that the index admission was used as the point at which disease-modifying therapy was actually started.

Similar crude mortality nevertheless requires a distinction that the literature has increasingly emphasized. In a prospective international multi-ethnic cohort, Lam et al. reported that after adjustment for age, sex, and clinical risk factors, patients with preserved EF had a lower risk of death than those with reduced EF (HR 0.62, 95% CI 0.46–0.85) [6]. Equal unadjusted mortality can therefore coexist with a genuine difference in EF-attributable risk, because the preserved-LVEF population carries a heavier burden of the factors that independently shorten survival. Our cohort is consistent with that reading, as follows: crude one-year mortality was identical although the preserved-LVEF group was older (median 82 vs. 79 years), more frail (73.5% vs. 59.4%), and more often affected by CKD (55.7% vs. 47.7%). We did not, however, reproduce an adjusted survival advantage for preserved EF. Among hospital survivors, the adjusted estimate favored the reduced-LVEF group, but only when NT-proBNP was included in the model, and it disappeared when NT-proBNP was omitted. Because natriuretic peptides are both a consequence of ventricular dysfunction and systematically higher at any given clinical severity when EF is reduced, adjusting for them removes part of the very risk that the phenotype is meant to capture. We therefore treat the adjusted LVEF coefficient cautiously and conclude that the 50% cut-off did not provide consistent prognostic information across model specifications.

### 4.4. Determinants of Post-Discharge Mortality

In-hospital death (27 events, 5.1%) and death after discharge (127 events over 12 months) differ by an order of magnitude in rate, and predictors identified during the index admission do not necessarily apply thereafter [7]. WRF, a strong marker of the severity of the acute episode, had no independent association with mortality once follow-up began at discharge (HR 1.04, *p* = 0.62) [12]. Cardiogenic shock could not even be estimated after discharge, because five of the six affected patients died before discharge. Frailty and admission NT-proBNP, by contrast, independently predicted mortality after discharge. This temporal separation may also explain the apparent paradox of a group with higher in-hospital mortality and equal mortality thereafter, as follows: the reduced-LVEF group loses patients early and predominantly to cardiovascular death, whereas the preserved-LVEF group loses them steadily after discharge.

Fitted separately within each phenotype among hospital survivors, none of the eight covariates showed heterogeneity (*p* for heterogeneity 0.072 to 0.987); the only nominally significant comparison, baseline eGFR (*p* = 0.045), does not survive correction for eight comparisons (Bonferroni-corrected *p* = 0.36). Model discrimination was nevertheless lower in the preserved-LVEF group (C-index 0.693) than in the LVEF < 50% group (0.751), suggesting that the conventional set of prognostic markers captures the risk of preserved EF less completely. A single prognostic model, of the kind exemplified by MAGGIC [13], therefore appears adequate for both phenotypes in the direction and magnitude of its coefficients, but not in its accuracy.

NT-proBNP is an integrated marker of congestion and wall stress with an established prognostic role across the EF spectrum [7], and frailty graded by the Clinical Frailty Scale is a powerful, bedside-applicable predictor of death in hospitalized HF [26] and in older patients with HF more generally [9,10,15]. Both retained their association after discharge in this cohort, whereas WRF did not, and cardiogenic shock could not be evaluated. These observations are consistent with the broader concept of multi-organ involvement in AHF, in which the number of organs affected on admission grades subsequent survival [27,28]; in our cohort the measurable organ-dysfunction signal was almost entirely renal, hepatic markers being only mildly deranged and differing little between phenotypes.

### 4.5. Limitations

This was a single-center observational study, and residual confounding cannot be excluded, particularly regarding the association between the preserved-LVEF phenotype and emergency-department utilization. The number of events constrained the multivariable model to approximately 10 candidate predictors, and one variable (NT-proBNP) was missing in 9 of the 503 patients discharged alive (1.8%), of whom 3 died; the primary models were therefore fitted by complete-case analysis, with multiple imputation yielding essentially identical estimates. The binary follow-up fields for non-fatal events were completed only for patients who remained alive and free of terminal RRT; consequently, they did not capture events preceding a subsequent death. For acute HF rehospitalization, however, event timing remained available through the composite time-to-event variables, allowing the sequence of events to be reconstructed; the competing-risks analysis and the composite endpoint are based on the whole cohort and are not subject to this bias. For emergency-department visits, no equivalent timing information exists, and those proportions remain conditional on survival. The worsening-renal-function gradient incorporates in-hospital RRT, which overlaps with the secondary endpoint, although it was used to predict the distinct outcome of all-cause mortality. Furthermore, data on discontinuation or down-titration of guideline-directed medical therapy in response to WRF were not systematically recorded, and such treatment changes could have influenced the observed outcomes. In addition, the post-discharge model is built from admission variables together with a small number of in-hospital changes; discharge NT-proBNP, which predicts post-discharge events better than admission NT-proBNP, was not systematically available, and time-varying covariates that change after discharge could not be modeled.

Several variables needed for a fuller phenotypic and pathophysiological characterization were not collected. Body weight and height were not recorded, so body mass index could not be reported and the obesity-related preserved-ejection-fraction phenotype could not be characterized; correspondingly, the use of glucagon-like peptide-1 receptor agonists or dual glucose-dependent insulinotropic polypeptide/glucagon-like peptide-1 receptor agonists in potentially eligible patients could not be assessed, and these agents were not in routine use for this indication at our institution during enrolment. Blood lactate was not measured systematically, precluding any analysis of hyperlactatemia, which is common in AHF even without overt peripheral hypoperfusion and identifies patients at high risk of adverse outcome [29]. No patient in the cohort required invasive mechanical ventilation, reflecting a registry conducted in cardiology wards and the coronary care unit rather than in a general intensive care unit. The use of non-invasive ventilatory support was not prospectively recorded and cannot be quantified, although acute pulmonary oedema was the presenting phenotype in 25.1% of patients; the requirement for inotropes or vasopressors was recorded and occurred in 11.5% of the cohort, with 1.1% presenting in cardiogenic shock. Clinical congestion was not quantified in a standardized way—jugular venous pressure, hepatomegaly, hepatojugular reflux, a third heart sound, and serial body weight were not scored prospectively—so the distinction between volume overload and fluid redistribution could be approached only indirectly, through the presenting phenotype and admission blood pressure, which was markedly higher in the preserved-LVEF group (150 vs. 140 mmHg, *p* < 0.001). No patient in the cohort had received cardiac resynchronization therapy, and 31 (5.8%) carried an implantable cardioverter-defibrillator, so device therapy could not be examined as a determinant of outcome. Cause of death could be adjudicated only for deaths occurring in-hospital; post-discharge mortality is reported as all-cause, so the contribution of competing non-cardiovascular causes to the convergence of one-year mortality cannot be quantified directly, although the in-hospital data show that cardiovascular death was concentrated in the reduced-LVEF group.

Our cohort was also older than those reported in many contemporary acute-HF registries (median age 81 years), which may limit direct comparability but reflects the aging reality of patients hospitalized with AHF in routine clinical practice.

## 5. Conclusions

Among patients hospitalized with AHF, preserved and reduced (LVEF < 50%) EF represent distinct clinical phenotypes that are dissimilar in in-hospital mortality and similar in post-discharge mortality, with no consistent independent contribution to prognosis of this specific cut-off of LVEF of 50%; the prognostic association of the 50% cut-off was not robust across model specifications. In-hospital death was more than twice as frequent with reduced EF and was almost exclusively cardiovascular, whereas after discharge the two phenotypes were indistinguishable. Post-discharge mortality was more strongly associated with natriuretic-peptide levels and frailty than with EF, WRF was not independently associated with post-discharge mortality, and cardiogenic shock predominantly characterized patients who died during the index hospitalization and could not be reliably evaluated among hospital survivors. Risk stratification in AHF should therefore extend beyond LVEF, incorporate markers of congestion, frailty and renal function, and specify the period over which risk is being predicted.

## Figures and Tables

**Table 1:** Baseline demographic and clinical characteristics by LVEF.

Parameter	Total (*n* = 530)	LVEF ≥ 50% (*n* = 264)	LVEF < 50% (*n* = 266)	*p*-Value
**Demographics**				
Male sex	284 (53.6%)	102 (38.6%)	182 (68.4%)	**<0.001**
Age (years)	81 (74–86)	82 (76–87)	79 (71–85)	**<0.001**
De novo heart failure	146 (27.5%)	69 (26.1%)	77 (28.9%)	0.531
Previous HF hospitalization (last 2y)	142 (26.8%)	65 (24.6%)	77 (28.9%)	0.305
**Frailty (Clinical Frailty Scale)**				
Frail (CFS ≥ 5)	352 (66.4%)	194 (73.5%)	158 (59.4%)	**<0.001**
**CFS category**				**<0.001**
CFS 1–3 (non-frail)	44 (8.3%)	13 (4.9%)	31 (11.7%)	
CFS 4–5 (vulnerable/mild)	295 (55.7%)	137 (51.9%)	158 (59.4%)	
CFS 6–9 (moderate–severe)	191 (36.0%)	114 (43.2%)	77 (28.9%)	
**Comorbidities**				
Ischemic etiology	167 (31.5%)	20 (7.6%)	147 (55.3%)	**<0.001**
Atrial fibrillation	313 (59.1%)	179 (67.8%)	134 (50.4%)	**<0.001**
**AF type**				**<0.001**
Paroxysmal	101 (19.1%)	47 (17.8%)	54 (20.3%)	
Permanent/persistent	184 (34.7%)	114 (43.2%)	70 (26.3%)	
Unknown	28 (5.3%)	18 (6.8%)	10 (3.8%)	
Diabetes mellitus	275 (51.9%)	131 (49.6%)	144 (54.1%)	0.341
Arterial hypertension	462 (87.2%)	237 (89.8%)	225 (84.6%)	0.098
Airway disease	169 (31.9%)	87 (33.0%)	82 (30.8%)	0.666
Chronic kidney disease	274 (51.7%)	147 (55.7%)	127 (47.7%)	0.082
Prior stroke/TIA	39 (7.4%)	21 (8.0%)	18 (6.8%)	0.721
Active malignancy	51 (9.6%)	27 (10.2%)	24 (9.0%)	0.747
Previous malignancy	36 (6.8%)	21 (8.0%)	15 (5.6%)	0.375
**Coronary Artery Disease**				<0.001
No	116 (21.9%)	54 (20.5%)	62 (23.3%)	
Yes	238 (44.9%)	75 (28.4%)	163 (61.3%)	
Undefined	176 (33.2%)	135 (51.1%)	41 (15.4%)	
Prior CABG	62 (11.7%)	20 (7.6%)	42 (15.8%)	**0.005**
Prior valve replacement	33 (6.2%)	22 (8.3%)	11 (4.1%)	0.069
ICD	31 (5.8%)	1 (0.4%)	30 (11.3%)	**<0.001**
Permanent pacemaker	51 (9.6%)	28 (10.6%)	23 (8.6%)	0.537
Right heart failure	226 (42.6%)	100 (37.9%)	126 (47.4%)	**0.034**
**Baseline functional status**				
**Baseline NYHA class**				**<0.001**
NYHA I	22 (4.2%)	8 (3.0%)	14 (5.3%)	
NYHA II	103 (19.4%)	35 (13.3%)	68 (25.6%)	
NYHA III	321 (60.6%)	168 (63.6%)	153 (57.5%)	
NYHA IV	84 (15.8%)	53 (20.1%)	31 (11.7%)	
**Clinical phenotype at presentation**				
Acute decompensated HF	389 (73.4%)	198 (75.0%)	191 (71.8%)	0.463
Acute pulmonary oedema	133 (25.1%)	64 (24.2%)	69 (25.9%)	0.726
Cardiogenic shock	6 (1.1%)	0 (0.0%)	6 (2.3%)	**0.030**
Isolated right HF	2 (0.4%)	2 (0.8%)	0 (0.0%)	0.248
**Valvular disease (moderate/severe)**				
Mitral regurgitation	227 (42.8%)	94 (35.6%)	133 (50.0%)	**0.001**
Tricuspid regurgitation	232 (43.8%)	131 (49.6%)	101 (38.0%)	**0.009**
Aortic stenosis	105 (19.8%)	55 (20.8%)	50 (18.8%)	0.632
Aortic regurgitation	67 (12.6%)	33 (12.5%)	34 (12.8%)	1.000
Mitral stenosis	22 (4.2%)	16 (6.1%)	6 (2.3%)	**0.048**
Multivalvular disease	185 (34.9%)	90 (34.1%)	95 (35.7%)	0.763

Preserved 50. versus reduced LVEF (<50%). Continuous variables: median (IQR) or mean ± SD; categorical: *n* (%). Abbreviations: AF, atrial fibrillation; CABG, coronary artery bypass grafting; CFS, Clinical Frailty Scale; HF, heart failure; ICD, implantable cardioverter-defibrillator; LVEF, left ventricular ejection fraction; NYHA, New York Heart Association; TIA, transient ischemic attack.

**Table 2:** Heart-failure pharmacotherapy by LVEF.

Parameter	Total (*n* = 530)	LVEF ≥ 50% (*n* = 264)	LVEF < 50% (*n* = 266)	*p*-Value
**Chronic (Home) HF Medication**				
ARNI	20 (3.8%)	5 (1.9%)	15 (5.6%)	**0.042**
ACEi/ARB	267 (50.4%)	153 (58.0%)	114 (42.9%)	**<0.001**
Beta-blocker	302 (57.0%)	147 (55.7%)	155 (58.3%)	0.607
MRA	114 (21.5%)	48 (18.2%)	66 (24.8%)	0.080
SGLT2i	108 (20.4%)	39 (14.8%)	69 (25.9%)	**0.002**
Furosemide	265 (50.0%)	134 (50.8%)	131 (49.2%)	0.794
Furosemide dose (mg)	10 (0–40)	20 (0–40)	0 (0–40)	0.431
Statin (any)	307 (57.9%)	145 (54.9%)	162 (60.9%)	0.192
**Discharge medication (survivors)**				
ARNI [*n* = 503]	55 (10.9%)	4 (1.6%)	51 (20.6%)	**<0.001**
ACEi/ARB [*n* = 503]	197 (39.2%)	120 (46.9%)	77 (31.2%)	**<0.001**
Beta-blocker [*n* = 503]	391 (77.7%)	179 (69.9%)	212 (85.8%)	**<0.001**
MRA [*n* = 503]	293 (58.3%)	126 (49.2%)	167 (67.6%)	**<0.001**
SGLT2i [*n* = 503]	323 (64.2%)	137 (53.5%)	186 (75.3%)	**<0.001**
Furosemide [*n* = 503]	446 (88.7%)	236 (92.2%)	210 (85.0%)	**0.017**
Furosemide dose (mg) [*n* = 503]	40 (40–80)	40 (40–80)	40 (40–60)	**0.005**
Statin (any) [*n* = 503]	331 (65.8%)	153 (59.8%)	178 (72.1%)	**0.005**

Categorical variables: *n* (%); furosemide dose: median (IQR). Discharge medication assessed in hospital survivors (*n* = 503). Abbreviations: ACEi, angiotensin-converting enzyme inhibitor; ARB, angiotensin receptor blocker; ARNI, angiotensin receptor–neprilysin inhibitor; HF, heart failure; LVEF, left ventricular ejection fraction; MRA, mineralocorticoid receptor antagonist; SGLT2i, sodium–glucose cotransporter-2 inhibitor.

**Table 3:** Admission laboratory and echocardiographic findings by LVEF.

Parameter	Total (*n* = 530)	LVEF ≥ 50% (*n* = 264)	LVEF < 50% (*n* = 266)	*p*-Value
**Admission Laboratory & Vitals**				
Admission systolic BP (mmHg)	145 (127–170)	150 (130–175)	140 (120–160)	**<0.001**
Baseline GFR (mL/min/1.73 m^2^)	58 (43–76.8)	55 (42–74)	62.5 (44–78.8)	**0.040**
Admission GFR (mL/min/1.73 m^2^)	57 (40–75)	54 (38–72)	59 (41–77)	0.134
Admission creatinine (mg/dL)	1.14 (0.92–1.53)	1.12 (0.88–1.50)	1.17 (0.95–1.58)	0.103
Admission urea (mg/dL)	56 (43–81.8)	57.5 (44–83.5)	55 (41.2–80)	0.167
NT-proBNP (pg/mL) [*n* = 519]	5248 (3032–10533)	4321 (2482–8344)	6900 (3850–14389)	**<0.001**
Troponin (pg/mL)	28.8 (14.3–69.4)	20.3 (11.2–38.9)	46.2 (22.6–114)	**<0.001**
CRP (mg/L)	0.90 (0.40–2.20)	0.80 (0.40–2.10)	1 (0.40–2.40)	0.202
WBC (×10^3^/µL)	8.65 (6.80–11.1)	8.60 (6.64–10.7)	8.70 (7.10–11.4)	0.207
Neutrophil-to-lymphocyte ratio	4.18 (2.86–6.43)	4.33 (3.17–6.55)	4 (2.62–6.26)	**0.014**
Albumin (g/dL) [*n* = 524]	4 (3.80–4.30)	4 (3.80–4.30)	4 (3.80–4.30)	0.769
Total protein (g/dL) [*n* = 514]	6.70 (6.30–7.10)	6.80 (6.20–7.10)	6.70 (6.30–7.10)	0.748
AST (IU/L)	27 (21–39)	26 (21–37)	28 (21–42)	0.059
ALT (IU/L)	19 (13–31)	18 (12–28)	21 (14–35)	0.003
GGT (IU/L)	31 (19–54)	28 (19–51)	34 (20–56)	0.166
ALP (IU/L)	79 (64–104)	81 (64–104)	77 (63–103)	0.453
Hemoglobin (g/dL)	11.7 (10.4–13)	11.4 (10.3–12.6)	12.1 (10.4–13.3)	**0.002**
RDW (%)	16.2 (15–18.3)	16.2 (15.3–18.6)	16.2 (14.7–17.9)	0.096
Sodium (mEq/L)	139 (137–141)	139 (137–141)	139 (137–141)	0.490
Potassium (mEq/L)	4.51 (4.14–4.91)	4.49 (4.16–4.86)	4.55 (4.13–4.94)	0.630
HbA1c (%) [*n* = 522]	6.10 (5.60–6.90)	6 (5.60–6.80)	6.20 (5.62–7)	**0.041**
Glucose (mg/dL)	132 (109–180)	132 (109–169)	132 (110–191)	0.302
Total cholesterol (mg/dL) [*n* = 527]	134 (110–161)	137 (109–162)	130 (110–159)	0.304
LDL (mg/dL) [*n* = 528]	73 (55–96.4)	74 (56.5–99.5)	71 (54–93)	0.533
HDL (mg/dL) [*n* = 527]	37 (30.4–46.7)	37.5 (30.8–48.1)	36.2 (30.3–44.8)	0.150
Triglycerides (mg/dL) [*n* = 528]	101 (77–130)	100 (78–132)	101 (77–127)	0.756
Ferritin (ng/mL) [*n* = 507]	54.7 (29.8–118)	51.8 (30.5–114)	56.9 (29.2–131)	0.368
TSH (µIU/mL) [*n* = 518]	1.22 (0.72–2.09)	1.25 (0.71–2.21)	1.17 (0.74–1.95)	0.392
Echocardiographic parameters				
LVEF (%)	45 (30–55)	55 (55–60)	30 (20–40)	**<0.001**
Estimated PASP (mmHg) [*n* = 529]	40 (35–55)	45 (35–60)	40 (30–50)	**<0.001**
E/e′ [*n* = 518]	16.5 (13–23)	16 (12–21)	18 (14–24)	**0.021**
RV S′ (cm/s) [*n* = 528]	10 (9–12)	11 (9–13)	10 (8.50–12)	**0.001**
TAPSE (mm) [*n* = 528]	18 (15–19)	18 (16–20)	17 (15–19)	**<0.001**
LAVi (mL/m^2^) [*n* = 528]	51 (43.8–63)	52 (43.5–64)	51 (44–63)	0.945
Pulmonary hypertension probability				**0.022**
Low	75 (14.2%)	32 (12.2%)	43 (16.2%)	
Intermediate	174 (32.8%)	76 (28.9%)	98 (36.8%)	
High	281 (53.0%)	156 (59.1%)	125 (47.0%)	
LV dilation	135 (25.5%)	12 (4.5%)	123 (46.2%)	**<0.001**
RV dilation	130 (24.5%)	64 (24.2%)	66 (24.8%)	0.979

Continuous variables: median (IQR) or mean ± SD; categorical: *n* (%). Available *n* is shown where values were missing. Abbreviations: AHF, acute heart failure; ALP, alkaline phosphatase; ALT, alanine aminotransferase; AST, aspartate aminotransferase; BP, blood pressure; CRP, C-reactive protein; E/e′, ratio of early mitral inflow to early diastolic mitral annular velocity; ED, emergency department; GFR, glomerular filtration rate; GGT, gamma-glutamyl transferase; HbA1c, glycated hemoglobin; HDL, high-density lipoprotein cholesterol; LAVi, left atrial volume index; LDL, low-density lipoprotein cholesterol; LV, left ventricular; LVEF, left ventricular ejection fraction; NT-proBNP, N-terminal pro–B-type natriuretic peptide; PASP, pulmonary artery systolic pressure; RDW, red cell distribution width; RV, right ventricular; TAPSE, tricuspid annular plane systolic excursion; TSH, thyroid-stimulating hormone; WBC, white blood cell count.

**Table 4:** In-hospital course and post-discharge outcomes by LVEF. Categorical comparisons use Fisher’s exact test.

Parameter	Total	LVEF ≥ 50%	LVEF < 50%	*p*-Value
**In-Hospital Course & Outcomes (*n* = 530; 264 vs. 266)**				
Need for inotropes/vasopressors	61 (11.5%)	26 (9.8%)	35 (13.2%)	0.290
Acute kidney injury	255 (48.1%)	124 (47.0%)	131 (49.2%)	0.661
RRT during hospitalization	13 (2.5%)	5 (1.9%)	8 (3.0%)	0.584
**Worsening renal function class (in-hospital)**				0.791
WRF 0 (<1.25× baseline Cr)	267 (50.4%)	129 (48.9%)	138 (51.9%)	
WRF 1 (1.25–1.5×)	133 (25.1%)	70 (26.5%)	63 (23.7%)	
WRF 2 (>1.5–2×)	90 (17.0%)	44 (16.7%)	46 (17.3%)	
WRF 3 (>2–3×)	25 (4.7%)	15 (5.7%)	10 (3.8%)	
WRF 4 (>3×)	2 (0.4%)	1 (0.4%)	1 (0.4%)	
WRF 5 (in-hospital RRT)	13 (2.5%)	5 (1.9%)	8 (3.0%)	
**Other in-hospital outcomes**				
Length of stay (days) [*n* = 503]	7 (5–9)	7 (5–9)	7 (5–9)	0.131
In-hospital mortality	27 (5.1%)	8 (3.0%)	19 (7.1%)	0.046
In-hospital cardiovascular death	20 (3.8%)	3 (1.1%)	17 (6.4%)	0.002
In-hospital non cardiovascular death	7 (1.3%)	5 (1.9%)	2 (0.8%)	0.284
**Post-discharge outcomes (*n* = 503; 256 vs. 247)**				
3-month mortality	35 (7.0%)	19 (7.4%)	16 (6.5%)	0.810
6-month mortality	68 (13.5%)	42 (16.4%)	26 (10.5%)	0.072
6-month ED visit [*n* = 422]	218 (51.7%)	116 (55.5%)	102 (47.9%)	0.142
6-month readmission (all-cause) [*n* = 422]	147 (34.8%)	83 (39.7%)	64 (30.0%)	**0.048**
6-month readmission for AHF [*n* = 422]	80 (19.0%)	45 (21.5%)	35 (16.4%)	0.226
**Mortality by period**				
In-hospital (*n* = 530)	27 (5.1%)	8 (3.0%)	19 (7.1%)	0.046
Post-discharge (*n* = 503)	127 (25.2%)	71 (27.7%)	56 (22.7%)	0.229
Total, one year (*n* = 530)	154 (29.1%)	79 (29.9%)	75 (28.2%)	0.702

Abbreviations: Cr: Creatinine; LVEF, Left ventricular ejection fraction; RRT, Renal replacement therapy; WRF, worsening renal function.

**Table 5:** Post-discharge clinical endpoints by LVEF.

Endpoint	Total (*n* = 503)	LVEF ≥ 50% (*n* = 256)	LVEF < 50% (*n* = 247)	*p*
**Primary & Secondary Endpoints (Patients Discharged Alive, *n* = 503)**				
All-cause death (primary)	127 (25.2%)	71 (27.7%)	56 (22.7%)	0.229
Terminal renal replacement therapy (secondary)	15 (3.0%)	7 (2.7%)	8 (3.2%)	0.944
Death or RRT (composite)	137 (27.2%)	76 (29.7%)	61 (24.7%)	0.247
Death, AHF rehospitalization, or RRT (composite)	231 (45.9%)	127 (49.6%)	104 (42.1%)	0.110
**Non-fatal events (patients alive and free of terminal RRT; *n* = 366–367)**				
All-cause readmission [*n* = 366]	164 (44.8%)	86 (47.8%)	78 (41.9%)	0.293
Readmission for acute HF [*n* = 366]	94 (25.7%)	51 (28.3%)	43 (23.1%)	0.282
Emergency department visit [*n* = 367]	234 (63.8%)	125 (69.1%)	109 (58.6%)	**0.048**

Abbreviations: AHF, acute heart failure; HF, heart failure; LVEF, left ventricular ejection fraction; RRT, renal replacement therapy. Data are *n* (%). Death (primary) and RRT (secondary) are cumulative incidences over the full post-discharge cohort of 503 hospital survivors; a patient reaching RRT is counted as an event irrespective of subsequent death, and patients who died without RRT are counted as non-events. Composite endpoints combine these by occurrence of any component. Non-fatal events were assessed only in patients alive and free of RRT through 12 months; the available *n* is shown. Groups compared by Fisher’s exact test.

**Table 6:** Post-discharge triple composite endpoint. Post-discharge follow-up ended 12 months after the index admission and therefore varied according to length of stay (death, acute HF rehospitalization, or renal replacement therapy) by LVEF category.

Parameter	Total (*n* = 503)	≥50% (*n* = 256)	<50% (*n* = 247)	*p*
**Composite Event**				
At 6 months	161 (32.0%)	92 (35.9%)	69 (27.9%)	0.068
By end of follow-up	231 (45.9%)	127 (49.6%)	104 (42.1%)	0.110
**Time-to-event through 12 months after admission**				
Event-free survival (KM)	51.6%	49.2%	54.0%	
HR, <50% vs. ≥50% (reference)	0.81 (0.63–1.05)			0.113
Log-rank				0.113
Competing-risks analysis				
First AHF rehospitalization, *n*	151	83	68	
CIF by end of follow-up	–	31.4%	25.6%	0.13
Subdistribution HR, <50% vs. ≥50%	0.78 (0.57–1.08)			0.13

Abbreviations: HF, heart failure; HR, hazard ratio; LVEF, left ventricular ejection fraction. The triple composite is the first occurrence of death, acute HF rehospitalization or RRT, reported as cumulative incidence over the full post-discharge cohort of 503 hospital survivors. Event rates compared by Fisher’s exact test; time-to-event by Kaplan–Meier (log-rank) and an unadjusted Cox model (LVEF ≥ 50% reference).

**Table 7:** Multivariable Cox models for post-discharge all-cause mortality, overall and stratified by LVEF category. Hazard ratios with 95% confidence intervals; time zero is discharge. Cardiogenic shock was not estimable, since only one of the six patients presenting in shock survived to discharge, and was therefore omitted. All three models were fitted in hospital survivors, so that every covariate is determined before the start of follow-up. *p* for heterogeneity is the Wald test of the difference between the corresponding coefficients in the two LVEF strata. Abbreviations: CFS, Clinical Frailty Scale; CI, confidence interval; eGFR, estimated glomerular filtration rate; HR, hazard ratio; LVEF, left ventricular ejection fraction; NT-proBNP, N-terminal pro–B-type natriuretic peptide; WRF, worsening renal function.

Covariate	Hospital Survivors (*n* = 494, 124 Deaths)	LVEF ≥ 50% (*n* = 252, 70 Deaths)	LVEF < 50% (*n* = 242, 54 Deaths)	*p* for Heterogeneity
LVEF < 50%	0.58 (0.38–0.89)	–	–	–
Age (per 10 years)	1.18 (0.90–1.55)	1.03 (0.70–1.50)	1.30 (0.85–1.98)	0.420
Male sex	1.51 (1.03–2.22)	1.95 (1.21–3.15)	0.98 (0.55–1.75)	0.072
Frailty (CFS ≥ 5)	1.96 (1.09–3.51)	1.98 (0.90–4.35)	1.96 (0.83–4.63)	0.987
Log NT-proBNP	1.86 (1.45–2.38)	2.15 (1.52–3.04)	1.67 (1.16–2.40)	0.324
Baseline eGFR (per 10 mL/min/1.73 m^2^)	0.97 (0.87–1.08)	1.07 (0.93–1.24)	0.85 (0.72–1.01)	0.045
Hemoglobin (g/dL)	0.99 (0.88–1.11)	1.03 (0.88–1.20)	0.99 (0.84–1.18)	0.758
Sodium(mEq/L)	1.00 (0.95–1.04)	1.00 (0.94–1.06)	0.98 (0.91–1.06)	0.679
WRF class (per grade)	1.04 (0.88–1.23)	0.97 (0.78–1.21)	1.20 (0.92–1.55)	0.234
C-index	0.710	0.693	0.751	–

## Data Availability

The data supporting the findings of this study are not publicly available due to ethical considerations and patient confidentiality. Access to the dataset may be provided upon reasonable request to the corresponding author, subject to approval by the Bioethics and Ethics Committee of the Venizelio General Hospital of Heraklion.

## Supplementary Materials

The following supporting information can be downloaded at https://www.mdpi.com/article/10.3390/jcdd13090423/s1, Table S1: Left ventricular ejection fraction (LVEF) categories and study grouping; Table S2: Chronic kidney disease (CKD) stages, KDIGO 2012 guidelines; Table S3: Clinical Frailty Scale (CFS) categories and descriptions; Table S4: Worsening renal function (WRF) class; Table S5: Clinical phenotypes of acute heart failure at presentation, 2021 ESC guidelines; Table S6: Echocardiographic probability of pulmonary hypertension (PH), 2022 ESC/ERS guidelines; Table S7: Sensitivity analyses of the association between LVEF and post-discharge mortality; Table S8: Prognostic contribution of individual clinical and echocardiographic substrates within the preserved-LVEF group.

## Abbreviations

The following abbreviations are used in this manuscript:

ACEi	Angiotensin-converting enzyme inhibitor
AF	Atrial fibrillation
AHF	Acute heart failure
AKI	Acute kidney injury
ALP	Alkaline phosphatase
ALT	Alanine aminotransferase
AST	Aspartate aminotransferase
ARB	Angiotensin receptor blocker
ARNI	Angiotensin receptor–neprilysin inhibitor
BP	Blood pressure
CABG	Coronary artery bypass grafting
CFS	Clinical Frailty Scale
C-index	Harrell concordance index
CI	Confidence interval
CRP	C-reactive protein
E/e′	Ratio of early mitral inflow to early diastolic mitral annular velocity
ED	Emergency department
EF	Ejection fraction
ESC	European Society of Cardiology
GFR	Glomerular filtration rate
GGT	Gamma-glutamyl transferase
HbA1c	Glycated hemoglobin
HDL	High-density lipoprotein cholesterol
HF	Heart failure
HFpEF	Heart failure with preserved ejection fraction
HFrEF	Heart failure with reduced ejection fraction
HR	Hazard ratio
ICD	Implantable cardioverter-defibrillator
IQR	Interquartile range
KDIGO	Kidney Disease: Improving Global Outcomes
LAVi	Left atrial volume index
LDL	Low-density lipoprotein cholesterol
LV	Left ventricular
LVEF	Left ventricular ejection fraction
MRA	Mineralocorticoid receptor antagonist
NT-proBNP	N-terminal pro–B-type natriuretic peptide
NYHA	New York Heart Association
PASP	Pulmonary artery systolic pressure
RDW	Red cell distribution width
RRT	Renal replacement therapy
RV	Right ventricular
SD	Standard deviation
SGLT2i	Sodium–glucose cotransporter-2 inhibitor
TAPSE	Tricuspid annular plane systolic excursion
TIA	Transient ischemic attack
TSH	Thyroid-stimulating hormone
WBC	White blood cell count
WRF	Worsening renal function
