# Supplementary material for: Two Phenotypes, One Prognosis: Period-Specific Mortality and Prediction of Post-Discharge Events in Acute Heart Failure
Source: J Cardiovasc Dev Dis. 2026 Sep 1;13(9):423. doi: 10.3390/jcdd13090423 (PMC13607643; doi:10.3390/jcdd13090423)
Supplement: Supplementary file 1 [file jcdd-13-00423-s001.zip › jcdd-4456144-supplementary.pdf]

Supplementary Material

Two Phenotypes, One Prognosis: Period-Specific Mortality and Prediction of Post-Discharge Events in Acute Heart Failure

Supplementary Data

Supplementary Table S1. Left ventricular ejection fraction (LVEF) categories and study grouping. The primary analysis dichotomized patients at an LVEF threshold of 50% into a preserved group (LVEF ≥50%, n=264) and a reduced or mildly reduced group (LVEF <50%, n=266, comprising HFmrEF and HFrEF). LVEF was measured by the Simpson biplane method [1]. HFrEF/HFmrEF/HFpEF = heart failure with reduced/mildly reduced/preserved ejection fraction.

**Category**	**LVEF (%)**	**n**
Reduced (HFrEF)	≤40	221
Mildly reduced (HFmrEF)	41–49	45
Preserved (HFpEF)	≥50	264

Supplementary Table S2. Chronic kidney disease (CKD) stages, KDIGO 2012 guidelines [2]. CKD was defined as eGFR <60 mL/min/1.73 m^2 persisting for ≥3 months. eGFR = estimated glomerular filtration rate.

**CKD stage**	**eGFR (mL/min/1.73 m^2)**	**Description**
G1	≥90	Normal or high
G2	60–89	Mildly decreased
G3a	45–59	Mildly to moderately decreased
G3b	30–44	Moderately to severely decreased
G4	15–29	Severely decreased
G5	<15	Kidney failure

Supplementary Table S3. Clinical Frailty Scale (CFS) categories and descriptions [3]. Study classification: non-frail (CFS 1–3), vulnerable/mildly frail (CFS 4–5) and moderately-to-severely frail (CFS 6–9); frailty was defined as CFS ≥5. CFS = Clinical Frailty Scale.

**Score**	**Category**	**Description**
1	Very fit	Robust, active, energetic and motivated; exercise regularly and are among the fittest for their age.
2	Well	No active disease symptoms but less fit than category 1; active occasionally.
3	Managing well	Medical problems well controlled; not regularly active beyond routine walking.
4	Vulnerable	Not dependent but symptoms limit activities; often report being slowed up.
5	Mildly frail	Evident slowing; dependent in instrumental activities of daily living (finances, transport, heavy housework).
6	Moderately frail	Need help with all outside activities and with housekeeping; difficulty with stairs.
7	Severely frail	Completely dependent for personal care but stable and not at high risk of dying within 6 months.

8	Very severely frail	Completely dependent, approaching the end of life; could not recover from a minor illness.
9	Terminally ill	Approaching the end of life (life expectancy <6 months).

Supplementary Table S4. Worsening renal function (WRF) class. WRF was graded as an ordinal severity class from the ratio of peak in-hospital to baseline serum creatinine; the most severe grade (class 5) denotes initiation of in-hospital renal replacement therapy (RRT). Percentages are of the full cohort (n=530). The distribution did not differ between LVEF groups (p=0.79). RRT = renal replacement therapy.

Class	Peak/baseline serum creatinine ratio	n (%)
0	<1.25×	267 (50.4%)
1	1.25–1.50×	133 (25.1%)
2	1.51–2.00×	90 (17.0%)
3	2.01–3.00×	25 (4.7%)
4	>3.00×	2 (0.4%)
5	In-hospital RRT	13 (2.5%)

Supplementary Table S5. Clinical phenotypes of acute heart failure at presentation, 2021 ESC guidelines. Classified according to the 2021 ESC Heart Failure Guidelines [4]. HF = heart failure; JVP = jugular venous pressure; RV = right ventricle; SBP = systolic blood pressure.

Phenotype	Key clinical features
Acute decompensated HF (ADHF)	Gradual worsening of pre-existing HF (dyspnea, oedema); hemodynamically stable; no hypoperfusion. Most common presentation.
Acute pulmonary oedema (APO)	Severe respiratory distress, orthopnea, low oxygen saturation, bilateral crackles; rapid onset; pulmonary congestion on imaging.
Isolated right heart failure	Predominant right-sided congestion (elevated JVP, peripheral oedema, hepatomegaly) without significant left-sided congestion; reduced RV systolic function.
Cardiogenic shock	Hypotension (SBP <90 mmHg or vasopressors required) with end-organ hypoperfusion (oliguria, elevated lactate, cold peripheries, altered mentation) despite adequate filling.

Supplementary Table S6. Echocardiographic probability of pulmonary hypertension (PH), 2022 ESC/ERS guidelines [5]. * Additional echocardiographic signs of PH from at least two different categories: RV/LV basal diameter ratio >1.0; flattening of the interventricular septum; RV outflow-tract acceleration time <105 ms and/or mid-systolic notching; early diastolic pulmonary regurgitation velocity >2.2 m/s; pulmonary artery diameter >25 mm; inferior vena cava diameter >21 mm with reduced inspiratory collapse; right atrial area >18 cm^2. TR = tricuspid regurgitation; RV = right ventricle; LV = left ventricle.

TR peak velocity (m/s)	Additional PH signs *	PH probability
≤2.8 or not measurable	No	Low
≤2.8 or not measurable	Yes	Intermediate
2.9–3.4	No	Intermediate
2.9–3.4	Yes	High

>3.4	Not required	High

Supplementary Table S7. Sensitivity analyses of the association between LVEF and post-discharge mortality, all fitted in the 494 hospital survivors with complete data (124 deaths) with time zero at discharge, except the multiple-imputation model, which used all patients discharged alive. The association of the 50% cut-off was not robust across model specifications, and no alternative cut-point was associated with mortality. CI = confidence interval; HR = hazard ratio; LVEF = left ventricular ejection fraction; NT-proBNP = N-terminal pro–B-type natriuretic peptide.

**Model**	**HR for LVEF <50% (95% CI)**	**p**
Full covariate set (primary, hospital survivors)	0.58 (0.38–0.89)	0.012
Same model, NT-proBNP omitted	0.88 (0.60–1.27)	0.49
Adjusted for age and sex only	0.84 (0.58–1.22)	0.37
Multiple imputation (m=10)	0.78 (0.53–1.13)	0.19
Alternative cut-point: LVEF <40%	0.79 (0.51–1.23)	0.30
Alternative cut-point: LVEF <45%	0.73 (0.47–1.13)	0.16
Alternative cut-point: LVEF <55%	0.84 (0.56–1.27)	0.41

Supplementary Table S8. Prognostic contribution of individual clinical and echocardiographic substrates within the preserved-LVEF group, restricted to hospital survivors with time zero at discharge (n=252 with complete data, 70 deaths). Hazard ratios for post-discharge all-cause mortality; adjusted models include age, frailty (CFS ≥5) and log NT-proBNP. No substrate other than a history of arterial hypertension retained an independent association after adjustment; that inverse association is very likely to reflect residual confounding rather than protection, since only 10.3% of the group had no such history and those patients presented with lower systolic blood pressure and a frailer overall profile. Percentages refer to the 252 patients with complete data. CFS = Clinical Frailty Scale; CI = confidence interval; HR = hazard ratio; LVEF = left ventricular ejection fraction; NT-proBNP = N-terminal pro–B-type natriuretic peptide; PH = pulmonary hypertension.

**Substrate**	**Prevalence**	**Univariable HR (95% CI)**	**p**	**Adjusted HR (95% CI)**	**p**
Significant valve disease (any moderate/severe)	75.4%	1.18 (0.68–2.06)	0.559	0.86 (0.49–1.52)	0.607
Multivalvular disease	33.7%	1.52 (0.94–2.44)	0.087	1.24 (0.76–2.02)	0.384
Moderate/severe tricuspid regurgitation	49.6%	1.13 (0.71–1.81)	0.601	0.97 (0.60–1.57)	0.905
High echocardiographic PH probability	59.1%	1.49 (0.91–2.45)	0.111	1.11 (0.67–1.84)	0.692

Atrial fibrillation	67.1%	0.92 (0.56–1.51)	0.749	0.89 (0.54–1.47)	0.655
Chronic kidney disease	55.6%	1.17 (0.73–1.88)	0.521	0.68 (0.40–1.14)	0.145
Diabetes mellitus	49.2%	1.17 (0.73–1.87)	0.515	1.36 (0.84–2.20)	0.205
Arterial hypertension	89.7%	0.45 (0.25–0.82)	0.009	0.42 (0.23–0.76)	0.004

References

1. Lang, R.M.; Badano, L.P.; Mor-Avi, V.; Afilalo, J.; Armstrong, A.; Ernande, L.; Flachskampf, F.A.; Foster, E.; Goldstein, S.A.; Kuznetsova, T.; et al. Recommendations for Cardiac Chamber Quantification by Echocardiography in Adults: An Update from the American Society of Echocardiography and the European Association of Cardiovascular Imaging. J. Am. Soc. Echocardiogr. 2015, 28, 1–39.e14, https://doi.org/10.1016/j.echo.2014.10.003.
2. Stevens, P.E.; Levin, A.; Kidney Disease: Improving Global Outcomes Chronic Kidney Disease Guideline Development Work Group Members. Evaluation and Management of Chronic Kidney Disease: Synopsis of the KDIGO 2012 Clinical Practice Guideline. Ann. Intern. Med. 2013, 158, 825–830, https://doi.org/10.7326/0003-4819-158-11-201306040-00007.
3. Rockwood, K.; Song, X.; MacKnight, C.; Bergman, H.; Hogan, D.B.; McDowell, I.; Mitnitski, A. A global clinical measure of fitness and frailty in elderly people. CMAJ 2005, 173, 489–495, https://doi.org/10.1503/cmaj.050051.
4. McDonagh, T.A.; Metra, M.; Adamo, M.; Gardner, R.S.; Baumbach, A.; Böhm, M.; Burri, H.; Butler, J.; Čelutkienė, J.; Chioncel, O.; et al. 2021 ESC Guidelines for the diagnosis and treatment of acute and chronic heart failure. Eur. Heart J. 2021, 42, 3599–3726, https://doi.org/10.1093/eurheartj/ehab368.
5. Humbert, M.; Kovacs, G.; Hoeper, M.M.; Badagliacca, R.; Berger, R.M.F.; Brida, M.; Carlsen, J.; Coats, A.J.S.; Escribano-Subias, P.; Ferrari, P.; et al. 2022 ESC/ERS Guidelines for the diagnosis and treatment of pulmonary hypertension. Eur. Heart J. 2022, 43, 3618–3731, https://doi.org/10.1093/eurheartj/ehac237.
